# Supplementary figures and images for: Immunogenic and Protective Properties of mRNA Vaccine Encoding Hemagglutinin of Avian Influenza A/H5N8 Virus, Delivered by Lipid Nanoparticles and Needle-Free Jet Injection
Source: Vaccines (Basel). 2025 Aug 21;13(8):883. doi: 10.3390/vaccines13080883 (PMC12389797; doi:10.3390/vaccines13080883)

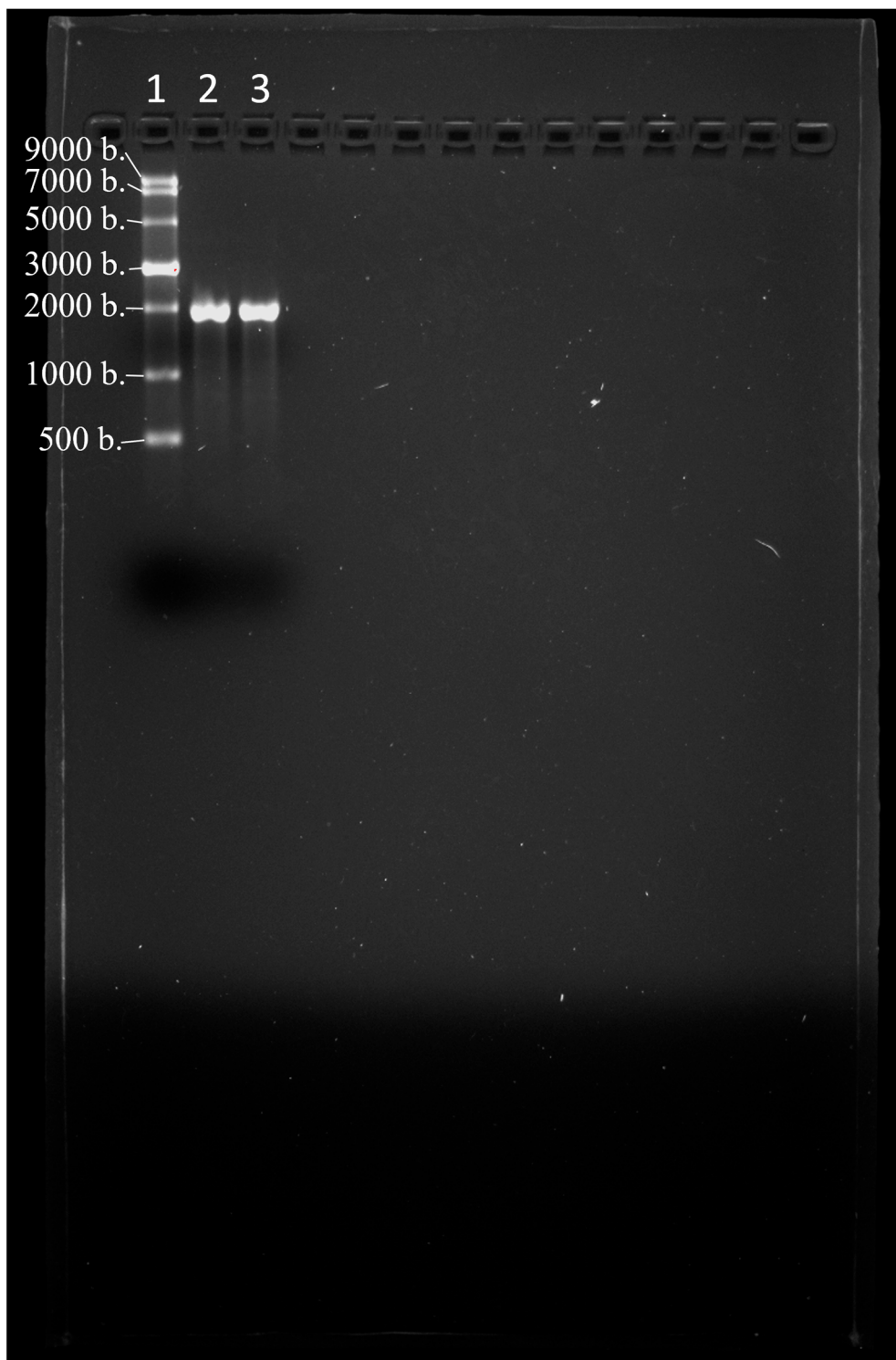

Supplement: Supplementary file 1 [file vaccines-13-00883-s001.zip › vaccines-3759486-supplementary.pdf]
